# CT radiomics for prediction of microvascular invasion in hepatocellular carcinoma: A systematic review and meta-analysis✰

**DOI:** 10.1016/j.clinsp.2023.100264

**Published:** 2023-08-08

**Authors:** Hai-ying Zhou, Jin-mei Cheng, Tian-wu Chen, Xiao-ming Zhang, Jing Ou, Jin-ming Cao, Hong-jun Li

**Affiliations:** a Medical Imaging Key Laboratory of Sichuan Province, and Department of Radiology, Affiliated Hospital of North Sichuan Medical College, Sichuan, China; b Department of Radiology, the Second Affiliated Hospital of Chongqing Medical University, Chongqing, China; c Department of Radiology, Nanchong Central Hospital/Second School of Clinical Medicine, North Sichuan Medical College, Sichuan, China; d Department of Radiology, Beijing YouAn Hospital, Capital Medical University, Beijing, China

**Keywords:** Radiomics, Microvascular invasion, Hepatocellular carcinoma, Computed tomography, Systematic review, Meta-analysis

## Abstract

The power of computed tomography (CT) radiomics for preoperative prediction of microvascular invasion (MVI) in hepatocellular carcinoma (HCC) demonstrated in current research is variable. This systematic review and meta-analysis aim to evaluate the value of CT radiomics for MVI prediction in HCC, and to investigate the methodologic quality in the workflow of radiomics research. Databases of PubMed, Embase, Web of Science, and Cochrane Library were systematically searched. The methodologic quality of included studies was assessed. Validation data from studies with Transparent Reporting of a Multivariable Prediction Model for Individual Prognosis or Diagnosis (TRIPOD) statement type 2a or above were extracted for meta-analysis. Eleven studies were included, among which nine were eligible for meta-analysis. Radiomics quality scores of the enrolled eleven studies varied from 6 to 17, accounting for 16.7%–47.2% of the total points, with an average score of 14. Pooled sensitivity, specificity, and Area Under the summary receiver operator Characteristic Curve (AUC) were 0.82 (95% CI 0.77–0.86), 0.79 (95% CI 0.75–0.83), and 0.87 (95% CI 0.84–0.91) for the predictive performance of CT radiomics, respectively. Meta-regression and subgroup analyses showed radiomics model based on 3D tumor segmentation, and deep learning model achieved superior performances compared to 2D segmentation and non-deep learning model, respectively (AUC: 0.93 vs. 0.83, and 0.97 vs. 0.83, respectively). This study proves that CT radiomics could predict MVI in HCC. The heterogeneity of the included studies precludes a definition of the role of CT radiomics in predicting MVI, but methodology warrants uniformization in the radiology community regarding radiomics in HCC.

## Highlights

• CT radiomics could preoperatively predict MVI in HCC with an AUC of 0.87.
• Radiomics model based on 3D tumor segmentation, and deep learning model can be superior to predict MVI.
• Reproducibility of current radiomics models for clinical application may be uncertain.

## Introduction

Hepatocellular Carcinoma (HCC) is the sixth most prevalent malignancy globally [1]. Although extensive efforts have been made in the surveillance and treatment of HCC, 5 years of recurrence after hepatic surgery still remains a major challenge [2]. Microvascular Invasion (MVI) has been considered an independent predictor of postoperative recurrence and poor prognosis after surgical hepatic resection. For the HCC patients with MVI, more aggressive treatment strategies, such as a wide resection margin, and preoperative neoadjuvant therapy, should be performed to improve survival through eradicating micro-metastases [3]. Hence, an assessment of MVI status before surgery is of great clinical relevance in HCC treatment decision-making. However, MVI is a histologic diagnosis based on postoperative microscopic examination of surgical specimens [4]. Preoperative prediction of MVI is still challenging [5]. Exploring new methods to preoperatively evaluate MVI status in HCC is of great importance.

As an emerging approach that can mine the hidden information in medical images to extract high-throughput imaging features and convert them into mineable data for quantitative analysis, radiomics has been also used to predict MVI in HCC and has shown the potential value for MVI prediction [6]. A number of radiomics models based on Computed Tomography (CT) data for MVI prediction have been constructed. However, as the methodologic variability in current CT radiomics research, such as the differences in imaging phase, model construction, sample size and so on, the diagnostic power of CT radiomics for preoperative evaluation of MVI remains variable in the reported studies 7, 8, 9, 10, 11, 12, 13, 14, 15, 16, 17. Hence, the authors searched relevant studies and performed this systematic review and meta-analysis to evaluate the value of CT radiomics for the MVI prediction in HCC, and to investigate the methodologic quality in the workflow of the radiomics research.

## Materials and methods

The study was registered prospectively in the International Prospective Register of Systematic Reviews (No.: CRD42022333822) and complied with the guidance of the Preferred Reporting Items for a Systematic Review and Meta-analysis of Diagnostic Test Accuracy Studies (PRISMA-DTA). Ethical approval and informed consent were waived because the present study did not collect patient information nor influence patient care.

### Literature research and study selection

All published relevant studies in English from the databases of PubMed, Embase, Web of Science, and Cochrane Library were systematically searched up to May 31, 2022. The search was performed according to the following terms: ((radiomics) OR (artificial Intelligence) OR (deep learning) OR (machine learning)) AND ((CT) OR (computed tomography)) AND ((hepatocellular carcinoma) OR (hepatoma) OR (hepatic tumor) OR (HCC) OR (liver cancer)) AND ((microvascular invasion) OR (MVI) OR (vascular invasion)). Reference lists of the included studies were also searched manually to recruit any potentially eligible studies.

After the removal of the publications in the form of letters, conference abstracts, editorials, reviews, case reports and duplicates, the studies which met the following criteria were included: 1) Patient population consisted of HCC patients with MVI confirmed by pathology after surgical resection or liver transplantation; 2) Radiomics based on CT images was performed for preoperative MVI prediction; and 3) The main result or one of the main results was the diagnostic accuracy of CT radiomics for predicting MVI in HCC. The authors excluded studies according to the following criteria: 1) Preoperative reception of antitumor therapy, such as systemic chemotherapy, transarterial chemoembolization, and radiofrequency ablation; 2) A two-by-two table could not be constructed from the data; 3) An animal experiment; or 4) The sample size of the study is less than 30.

All identified articles were first screened by title and abstract, and then full-text reviews of potentially eligible articles were performed independently by two authors (the first and the second authors with 12 and 3 years of radiological experience, respectively). Any disagreement was resolved by discussion to reach a consensus. Reference lists of the included studies were also searched manually to recruit any potentially eligible studies.

### Data extraction

The following information was extracted from each paper by two authors in consensus (the first and the third authors with 12 and 23 years of radiological experience, respectively): 1) Study characteristics including authors, year of publication, study type, study design, and study country; 2) Subject characteristics including the total number of participants, the MVI-positive and MVI-negative cases, sensitivity, specificity and Area Under the receiver operator Characteristic Curve (AUC). The number of True Positives (TPs), False Positives (FPs), False Negatives (FNs), and True Negatives (TNs) were calculated by using the above-mentioned information in each included study; 3) Radiomics model characteristics including imaging phase, region segmentation, feature selection, clinical features, radiological features, modeling method, and validation method. In studies the data were split into training and validation cohorts, only validation data of type 2a or above according to Transparent Reporting of a Multivariable Prediction Model for Individual Prognosis or Diagnosis (TRIPOD) statement [18] were extracted for meta-analysis to avoid the potential bias from training processes of radiomics models in the training cohorts. If there were two or more radiomics models based on the same group of patients in one study, the model with the best diagnostic performance was included in the present meta-analysis.

### Assessment of radiomics quality score and study quality

The previous two reviewers (the first and the third authors) assessed the methodologic quality of the included literature in consensus by a scoring system proposed by Lambin in 2017 – the Radiomics Quality Score (RQS), according to 6 domains with 16 items [19]. Domain 1 assesses the quality and reproducibility of image and segmentation; domain 2, the reporting of feature reduction and validation; domain 3, biological validation and clinical utility; domain 4, model performance; domains 5 and 6, demonstration of high level of evidence and open science, respectively. The ideal score of the RQS is 36 points, corresponding to a percentage of 100%. The Quality Assessment of Diagnostic Accuracy Studies (QUADAS-2) tool was also used to evaluate the risk of bias and concern of application in the four domains including patient selection, index test, reference standard, and flow and timing [20]. The results of each domain were categorized as yes, no or unclear for the risk of bias, and low risk, high risk, or unclear for applicability concerns.

### Statistical analysis

The pooled sensitivity, specificity, Positive Likelihood Ratio (PLR), and Negative Likelihood Ratio (NLR) were calculated. Then a Summary Receiver Operating Characteristic (SROC) curve was drawn, and the Area Under the SROC Curve (AUC) was used to evaluate the diagnostic power of the included studies on MVI prediction. An AUC of more than 0.9 indicated a high diagnostic value, while values between 0.7 to 0.9 and less than 0.7 indicated moderate and low diagnostic value, respectively.

Forest plots were drawn, and *I^2^* was considered to detect the heterogeneity among the included studies. *I*^2^ > 50% was regarded as substantial heterogeneity. To investigate the potential sources of heterogeneity, meta-regression and subgroup analysis of several relevant covariates were performed according to the imaging phase, region segmentation (3D or 2D), algorithm for feature extraction and selection (deep learning or non-deep learning), combined clinical features or radiological features (yes or no), and modeling method (deep learning or non-deep learning). Additionally, Deeks’ funnel plot and Deeks’ asymmetry test were performed to assess the publication bias.

All statistical analyses were carried out with Meta-DiSc version 1.4 and STATA version 16.0 (StataCorp LP, College Station, TX, USA).

## Results

### Literature selection and general characteristics of the included studies

**Fig. 1:**
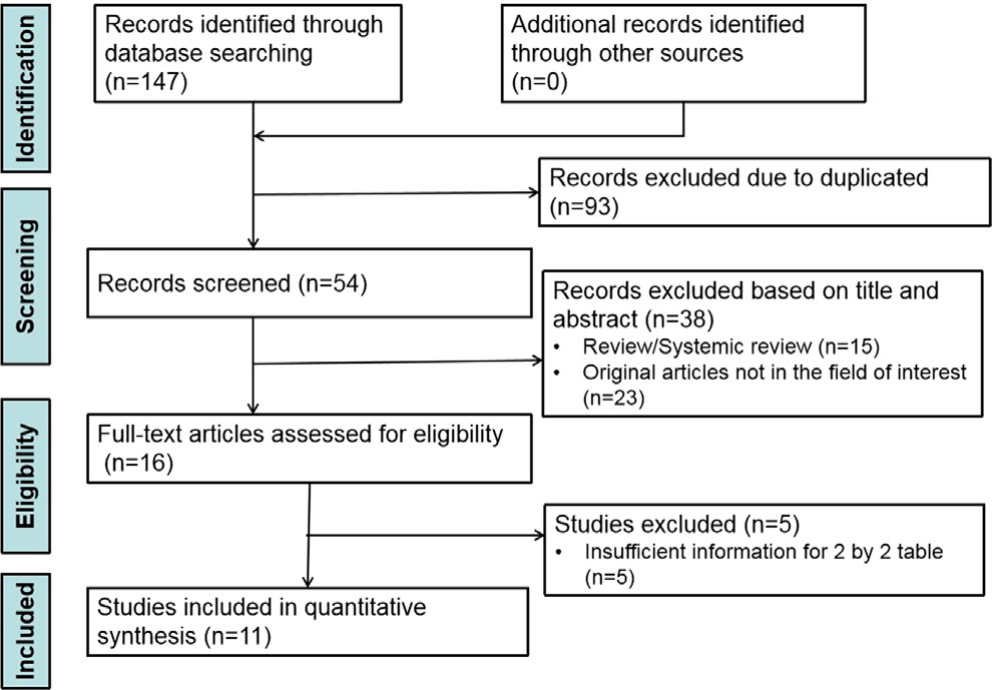
Flowchart of study selection.

The study selection procedure is depicted systematically in Fig. 1. In total, 11 studies published between April 2018 and May 2022, with 63.6% (7/11) within the three years (2020–2022) 7, 8, 9, 10, 11, 12, 13, 14, 15, 16, 17, were eligible for this systematic review and meta-analysis. 3298 HCC patients with 1344 (40.8%) MVI-positive and 1954 (59.2%) MVI-negative were studied. More details about the general characteristics of the included studies are shown in Table 1.

**Table 1:** General characteristics of the included studies.

Authors	Year	Study type	Study design	Study country	Number of tumors (MVI+/MVI-)	Phase	Region segmentation	Algorithm for feature selection	Combined clinical features	Combined radiological features	Modeling methods	Validation	TRIPOD type	Internal validation cohort (MVI+/MVI-)
Yao W, et al. [7].	2022	Retro	Single center	China	82 (49/33)	NC, AP, PVP, EP	2D Manual	Logistic regression	Yes	No	Logistic regression	N/A	Type 1a	N/A
Yang Y, et al. [8].	2022	Retro	Single center	China	283 (36/247)	NC, AP, PVP	3D Manual	SVM	Yes	Yes	CNN	Internal validation	Type 2a	85 (11/74)
Zhang W, et al. [9].	2021	Retro	Single center	China	111 (57/54)	PVP	3D Semiautomatic	N/A	No	No	Random forest	Internal validation	Type 2a	23 (10/13)
Liu SC, et al. [10].	2021	Retro	Multi- center	China	473 (135/338)	AP	2D Automatic	CNN	Yes	No	CNN	External validation	Type 3	93 (28/65)
Jiang YQ, et al. [11].	2021	Retro	Single center	China	405 (220/185)	AP, PVP, DP	3D Manual	3DCNN	No	Yes	3DCNN	Internal validation	Type 2a	81(44/37)
He M, et al. [12].	2020	Retro	Single center	China	145 (99/46)	PVP	3D Manual	LASSO	Yes	No	Logistic regression	Internal validation	Type 2a	44 (28/16)
Zhang X, et al. [13].	2020	Retro	Two centers	China	637 (255/382)	DP	2D Manual	LASSO	Yes	No	Logistic regression	External validation	Type 3	111 (43/68)
Ni M, et al. [14].	2019	Retro	Single center	China	206 (88/118)	PVP	2D Manual	LASSO	No	No	LASSO, GBDT	N/A	Type 1a	N/A
Xu X, et al. [15].	2019	Retro	Single center	China	495 (149/346)	AP, PVP	3D Semiautomatic	SVM	Yes	Yes	SVM	Internal validation	Type 2a	145 (49/96)
Ma X, et al. [16].	2019	Retro	Single center	China	157 (55/102)	PVP	3D Manual	LASSO	Yes	No	Logistic regression	Internal validation	Type 2a	47 (18/29)
Peng J, et al. [17].	2018	Retro	Single center	China	304 (201/103)	AP, PVP	2D Semiautomatic	LASSO	Yes	Yes	Logistic regression	Internal validation	Type 2a	120 (74/46)

MVI, Microvascular Invasion; TRIPOD, Transparent Reporting of a Multivariable Prediction Model for Individual Prognosis or Diagnosis; Retro, Retrospective. NC, Non-Contrast scan; AP, Arterial Phase; PVP, Portal Venous Phase; EP, Equilibrium Phase; DP, Delayed Phase; LASSO, Least Absolute Shrinkage and Selection Operator; SVM, Support Vector Machine; GBDT, Gradient Boosting Decision Tree; CNN, Convolutional Neural Network; N/A, Not Available.

### Radiomics model characteristics

The radiomics model characteristics are summarized as follows according to the typical workflow of radiomics research (Table 1).

### Imaging acquisition

In studies based on CT data, nine of them (9/11) applied the enhancement phases, including the Arterial Phase (AP) (1/9), Portal Venous Phase (PVP) (4/9), Delayed Phase (DP) (1/9), and the combination of them (3/9), while two studies applied both the unenhanced and enhanced scans.

### Region segmentation

Among the 11 enrolled studies, 3D and 2D tumor segmentation was performed in 6 and 5 studies, respectively. The six studies performed by 3D tumor segmentation included manual segmentation in four studies (4/6) and semiautomatic segmentation in two (2/6). The 2D segmentation was performed on the axial slice with the largest tumor diameter in the remaining five studies (5/11). The previous 2D segmentation was drawn manually in three studies (3/5), semi-automatically in one study (1/5), and automatically in one study (1/5).

### Feature extraction and selection

In the included studies, the most commonly used algorithm for feature extraction and selection was Least Absolute Shrinkage and Selection Operator (LASSO) regression (5/11), followed by Convolutional Neural Network (CNN) (2/11) and support vector machine (2/11).

### Modeling

Eight studies (8/11) constructed a non-deep learning model, in most of which (5/8) logistic regression was performed; and the remaining three (3/11) studies constructed a deep learning model with CNN or 3D CNN. The clinical risk factors and/or radiological features were used to construct a combined prediction model in 9/11 studies, among which five studies included clinical risk factors, one study included radiological features, and three studies included both of them. The commonly used clinical and radiological features included Hepatitis B Surface Antigen (HBsAg) or Hepatitis C Virus Antibody (HCVAb) status, Alpha-Fetoprotein (AFP), Child-Pugh score, Aspartate Aminotransferase (AST), tumor size, non-smooth tumor margin, ill-defined pseudo-capsule, peritumoral arterial enhancement, and portal vein tumor thrombosis.

### Validation method

In the enrolled 11 studies, the research subjects were randomly divided into a training cohort and an internal validation cohort at a certain ratio in seven studies, and into the training cohort and the internal and external validation cohorts in two studies. And the remaining two studies had no validation.

### RQS and risk of bias assessment

The RQS scores of the 11 studies varied from 6 to 17, accounting for 16.7%–47.2% of the total points, with an average score of 14 (14/36, 38.9%). In 9 of the 11 studies, the scores were credited between 14 (14/36, 38.9%) and 17 (17/36, 47.2%) points, and the remaining two studies achieved a point less than 10 (6/36 (16.7%), and 7/36 (19.4%)). The results of each included study are provided in Supplementary Table 1.

The items of image protocol, multiple segmentation, feature reduction, cut-off analyses, comparison with the gold standard, and discrimination statistics were performed in all the studies. In nine of the 11 studies, a validation test was performed, but only two of the nine studies applied an external validation and assigned 3 points. The remaining two studies (2/11) had no validation and were assigned –5 points. Due to the lack of prospective studies, deficiency of phantom studies on all scanners, absence of imaging at multiple time points, insufficiency of biological correlated discussion, shortness of cost-effectiveness analysis, and unavailable open science and data, all the 11 included studies obtained the point of zero in these items.

**Fig. 2:**
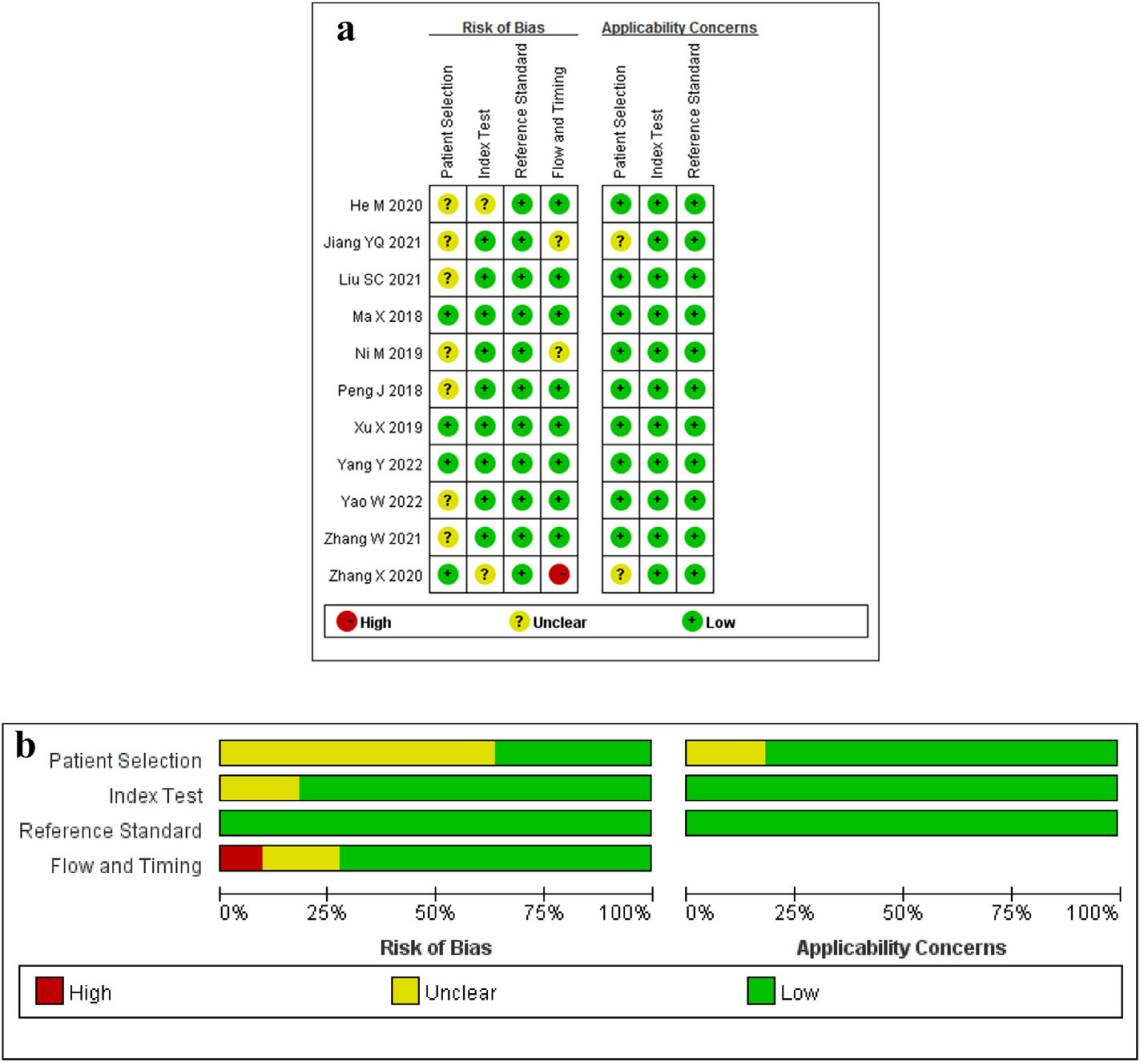
Methodologic quality assessment of the included studies based on the QUADAS-2 scale. (a) Individual studies, and (b) summary.

The results of the risk of bias and the applicability concerns assessed by the QUADAS-2 tool are shown in Fig. 2. A majority of studies showed a low or unclear risk of bias in each domain.

### Publication bias

**Fig. 3:**
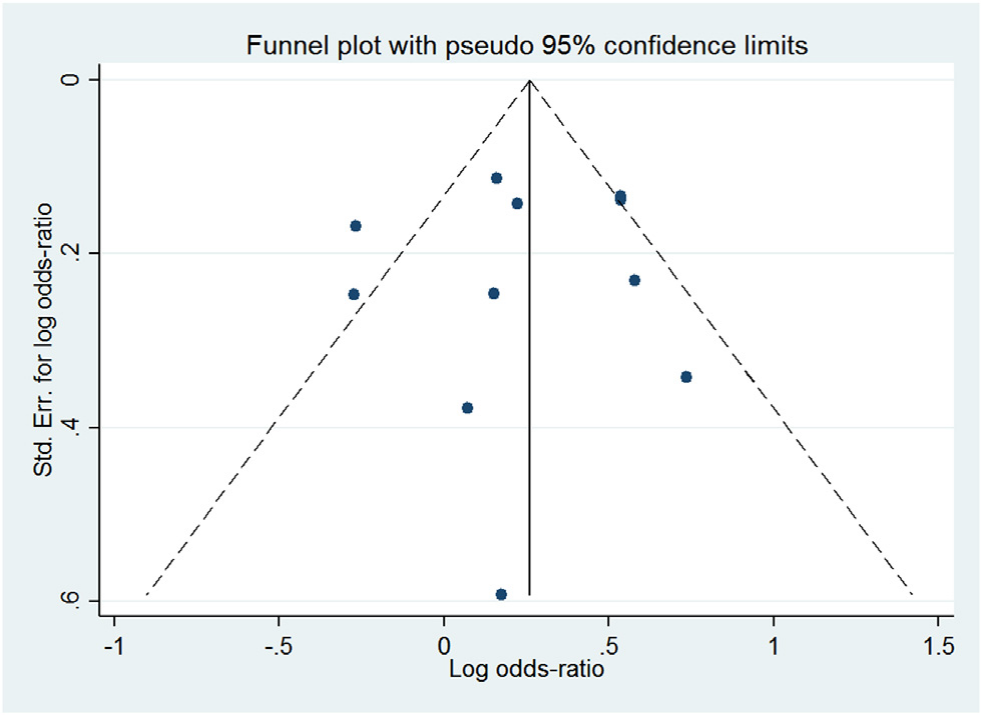
Deeks’ funnel plot. The plot shows no asymmetry and presence of publication bias.

Deeks’ funnel plot (Fig. 3) showed that the slope coefficients were relatively symmetrical (*p* > 0.05), suggesting that the publication bias of the included studies was not present.

### Meta-analysis

**Fig. 4:**
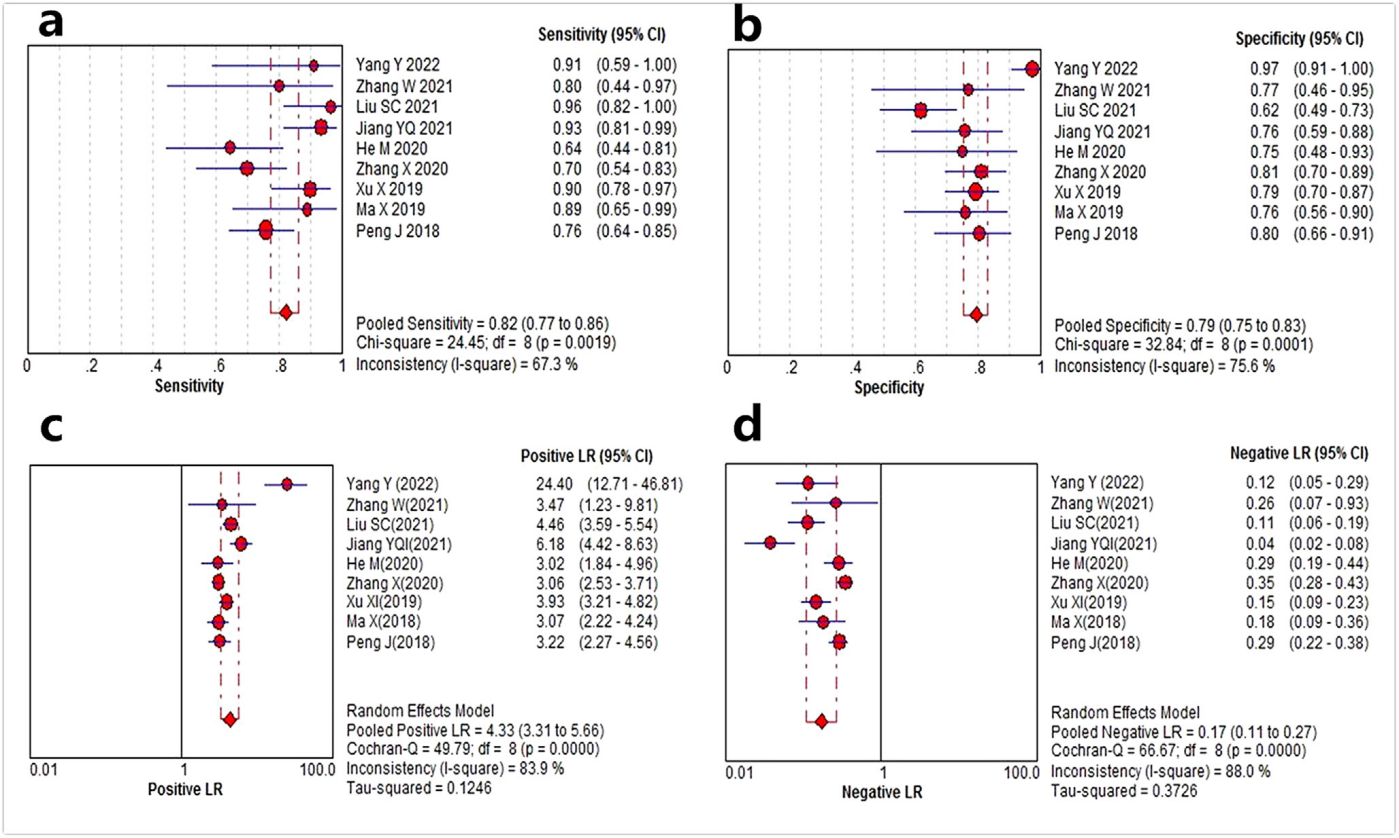
Forest plots show the performance estimates of each study with computed tomography radiomics for preoperative microvascular invasion evaluation in hepatocellular carcinoma. (a) Sensitivity, (b) Specificity, (c) Positive Likely Ratio (LR), and (d) Negative LR.

**Fig. 5:**
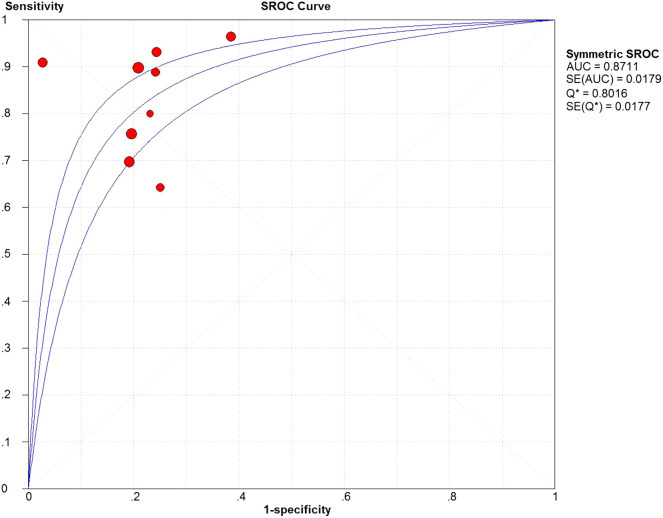
Summary Receiver Operating Characteristic (SROC) curve of computed tomography radiomics for preoperative microvascular invasion evaluation in hepatocellular carcinoma.

Data from 9 studies[8–13,15–17] with TRIPOD type 2a or above were analyzed to assess the value of CT radiomics models for the prediction of MVI in HCC. The forest plots of the 9 included studies are shown in Fig. 4. The pooled sensitivity, specificity, PLR and NLR for preoperative MVI evaluation were 0.82 (95% CI: 0.77–0.86, *I^2^* = 67.3%), 0.79 (95% CI: 0.75–0.83, *I^2^* = 75.6%), 4.33 (95% CI: 3.31–5.66, *I^2^* = 83.9%), and 0.17 (95% CI: 0.11–0.27, *I^2^* = 88.0%), respectively. The AUC based on the SROC was 0.87 (95% CI: 0.84–0.91) (Fig. 5).

### Meta-regression and subgroup analyses

As substantial heterogeneity among the included studies was suggested by the *I^2^* values of sensitivity, specificity, PLR, and NLR (all *I^2^* > 50%), the meta-regression analysis was performed. The results showed that region segmentation (3D or 2D), and modeling method (deep learning or non-deep learning) contributed to the study heterogeneity (*p*= 0.017 and 0.002, respectively).

The results of subgroup analyses are shown in Table 2. In terms of region segmentation, the pooled sensitivity, specificity, and AUC were higher in studies with 3D region segmentation than with 2D. The predictive model constructed by deep learning showed a higher diagnostic power than that by non-deep learning.

**Table 2:** Subgroup analyses of CT radiomics for preoperative MVI prediction in HCC.

**Parameter**	**Category**	No. **of studies**	**Pooled Sen (95% CI)**	**Pooled Spe (95% CI)**	**Pooled PLR (95% CI)**	**Pooled NLR (95% CI)**	**AUC (95% CI)**
Region segmentation	3D	6	0.90 (0.87–0.92)	0.83 (0.80–0.85)	5.05 (3.19–8.01)	0.14 (0.07–0.27)	0.93 (0.86–0.99)
	2D	3	0.79 (0.76–0.82)	0.77 (0.74–0.80)	3.55 (2.73–4.62)	0.24 (0.14–0.40)	0.83 (0.80–0.86)
Modeling method	Deep learning	3	0.94 (0.91–0.96)	0.86 (0.83–0.88)	8.16 (3.95–16.86)	0.08 (0.04–0.16)	0.97 (0.93–1.00)
	Non-deep learning	6	0.79 (0.76–0.82)	0.76 (0.73–0.79)	3.34 (2.98–3.76)	0.25 (0.19–0.34)	0.83 (0.80–0.85)

MVI, Microvascular Invasion; HCC, Hepatocellular Carcinoma; 95% CI, 95% Confidence Interval; Sen, Sensitivity; Spe, Specificity; PLR, Positive Likelihood Ratio; NLR, Negative Likelihood Ratio; AUC, Area Under the summary receiver operator Characteristic Curve.

## Discussion

The present study showed CT radiomics could be an efficient method to preoperatively predict MVI in HCC, with an AUC of 0.87. Radiomics models based on 3D region segmentation and deep learning achieved superior performances compared to 2D segmentation and non-deep learning, respectively. However, the methodologic quality of the included literature was insufficient.

Because of its high availability and low cost, CT is widely used for HCC examination. Although conventional CT features represent relatively few metrics for MVI prediction in HCC [21], CT radiomics could transform raw images into numerable quantitative features, and interpret tumor instinct pathophysiology. Thus, CT radiomics provides more possibility for MVI prediction [19,22]. However, due to the variability of imaging phases performed in the included studies, the numbers of studies with each imaging phase were relatively small in the present meta-analysis, and subgroup analysis could not be performed to evaluate the prediction power of CT radiomics models based on each phase, and the best phase for MVI prediction in radiomics research could not be recommended.

Given that the radiomics workflow involves multiple steps and that each step can be performed by several different strategies and approaches [6,19], the heterogeneity among the included radiomics studies was high in the present meta-analysis. Meta-regression analysis demonstrated that region segmentation (3D or 2D), and modeling method (deep learning or non-deep learning) contributed to the study heterogeneity in CT radiomics. The radiomics model based on 3D tumor segmentation achieved a superior performance for MVI prediction compared to 2D segmentation. The probable reason is that the volumes of interest derived from 3D tumor segmentation can provide the entire volumetric imaging features of the tumor and might be less influenced by hand-related artifacts. Meanwhile, the deep learning model was demonstrated to have a higher prediction power than the non-deep learning model. As a promising technique to learn features associated with a predefined task, deep learning has an advantage in learning features from the raw images without precise annotations, and in the learning process, feature extraction is not required, which avoids defects in human-designed features in radiomics analysis. Compared with the non-deep learning methods used in radiomics analysis, it needs less manpower and time for MVI prediction, and it is proven to be more powerful in various challenging clinical tasks [23,24]. Hence, it has been expected to improve the efficiency and reliability of constructed models.

Despite the promising results of the present meta-analysis, the overall methodologic quality of the included literature was insufficient, reducing the reliability and repeatability of the radiomics models for clinical implementation. The lack of prospective studies, deficiency of phantom studies on all scanners, absence of imaging at multiple time points, insufficiency of biological correlates discussion, shortness of cost-effectiveness analysis, and unavailable open science and data attributed to the low RQS scores. Moreover, although internal validation was performed in most studies, independent external validation was lacking. In the future, RQS should not only be used to assess the methodologic quality of radiomics research but also to guide the radiomics study design and should be used even as a routine self-checklist before manuscript submission.

This systematic review and meta-analysis have several limitations. Firstly, all included studies were designed retrospectively, which may cause a patient selection bias. Secondly, due to the numbers of studies with each CT imaging phase being relatively small, the best CT phase for MVI prediction in radiomics research could not be recommended. Thirdly, the authors did not perform the study on the validation of CT radiomics for the prediction of MVI in HCC to assess prognosis and treatment because the aims of this systematic review and meta-analysis were to evaluate the value of CT radiomics for the MVI prediction in HCC, and to investigate the methodologic quality in the workflow of the radiomics research.

In conclusion, the systemic review and meta-analysis demonstrate that CT radiomics could be an efficient method for preoperative MVI prediction in HCC. The radiomics model based on 3D tumor segmentation and deep learning could achieve superior performances compared to 2D segmentation and non-deep learning, respectively. However, the heterogeneity of the included studies precludes a definition of the role of CT radiomics in predicting MVI. It is necessary to design prospective studies with an external validation cohort in accordance with a standardized radiomics workflow and RQS items in the future to enhance the reliability and reproducibility of the radiomics models for clinical application.

## Authors’ contributions

Concept and Design: Hai-ying Zhou, Tian-wu Chen, Xiao-ming Zhang, Hong-jun Li. Data Collection and Processing: Hai-ying Zhou, Jin-mei Cheng, Tian-wu Chen. Analysis: Hai-ying Zhou, Jin-mei Cheng, Tian-wu Chen, Xiao-ming Zhang, Jing Ou, Jin-ming Cao. Manuscript Preparation and Editing: All authors.

## Declaration of Competing Interest

The authors declare no conflicts of interest.

## Appendix Supplementary materials

Image, application 1

## References

[1] Forner A., Reig M., Bruix J. (2019). Hepatocellular carcinoma. Lancet.

[2] Marrero J.A., Kulik L.M., Sirlin C.B., Zhu A.X., Finn R.S., Abecassis M.M. (2018). Diagnosis, staging, and management of hepatocellular carcinoma: 2018 practice guidance by the. Hepatology.

[3] Erstad D.J., Tanabe K.K. (2019). Prognostic and therapeutic implications of microvascular invasion in hepatocellular carcinoma. Ann Surg Oncol.

[4] Rastogi A. (2018). Changing role of histopathology in the diagnosis and management of hepatocellular carcinoma. World J Gastroenterol.

[5] Reginelli A., Vacca G., Segreto T., Picascia R., Clemente A., Urraro1 F. (2018). Can microvascular invasion in hepatocellular carcinoma be predicted by diagnostic imaging? A critical review. Future Oncol.

[6] Lv K., Cao X., Du P., Fu J.Y., Geng D.Y., Zhang J. (2022). Radiomics for the detection of microvascular invasion in hepatocellular carcinoma. World J Gastroenterol.

[7] Yao W., Yang S., Ge Y., Fan W., Xiang L., Wan Y. (2022). Computed tomography radiomics-based prediction of microvascular invasion in hepatocellular carcinoma. Front Med (Lausanne).

[8] Yang Y., Zhou Y., Zhou C., Ma X. (2022). Deep learning radiomics based on contrast enhanced computed tomography predicts microvascular invasion and survival outcome in early-stage hepatocellular carcinoma. Eur J Surg Oncol.

[9] Zhang W., Yang R., Liang F., Liu G., Chen A., Wu H. (2021). Prediction of microvascular invasion in hepatocellular carcinoma with a multi-disciplinary team-like radiomics fusion model on dynamic contrast-enhanced computed tomography. Front Oncol.

[10] Liu S.C., Lai J., Huang J.Y., Cho C.F., Lee P.H., Lu M.H. (2021). Predicting microvascular invasion in hepatocellular carcinoma: a deep learning model validated across hospitals. Cancer Imaging.

[11] Jiang Y.Q., Cao S.E., Cao S., Chen J.N., Wang G.Y., Shi W.Q. (2021). Preoperative identification of microvascular invasion in hepatocellular carcinoma by XGBoost and deep learning. J Cancer Res Clin.

[12] He M., Zhang P., Ma X., He B., Fang C., Jia F. (2020). Radiomic feature based predictive model for microvascular invasion in patients with hepatocellular carcinoma. Front Oncol.

[13] Zhang X., Ruan S., Xiao W., Shao J., Tian W., Liu W. (2020). Contrast-enhanced CT radiomics for preoperative evaluation of microvascular invasion in hepatocellular carcinoma: a two-center study. Clin Transl Med.

[14] Ni M., Zhou X., Lv Q., Li Z., Gao Y., Tan Y. (2019). Radiomics models for diagnosing microvascular invasion in hepatocellular carcinoma: which model is the best model?. Cancer Imaging.

[15] Xu X., Zhang H.L., Liu Q.P., Sun S.W., Zhang J., Zhu F.P. (2019). Radiomic analysis of contrast-enhanced CT predicts microvascular invasion and outcome in hepatocellular carcinoma. J Hepatol.

[16] Ma X., Wei J., Gu D., Zhu Y., Feng B., Liang M. (2019). Preoperative radiomics nomogram for microvascular invasion prediction in hepatocellular carcinoma using contrast-enhanced CT. Eur Radiol.

[17] Peng J., Zhang J., Zhang Q., Xu Y., Zhou J., Liu L. (2018). A radiomics nomogram for preoperative prediction of microvascular invasion risk in hepatitis B virus-related hepatocellular carcinoma. Diagn Interv Radiol.

[18] Collins G.S., Reitsma J.B., Altman D.G., Moons K.G. (2015). Transparent reporting of a multivariable prediction model for individual prognosis or diagnosis (TRIPOD): the TRIPOD statement. BMJ.

[19] Lambin P., Leijenaar R.T.H., Deist T.M., Peerlings J., de Jong E.E.C., Timmeren J. (2017). Radiomics: the bridge between medical imaging and personalized medicine. Nat Rev Clin Oncol.

[20] Whiting P.F., Rutjes A.W.S., Westwood M.E., Mallett S., Deeks J.J., Reitsma J.B. (2011). QUADAS-2: a revised tool for the quality assessment of diagnostic accuracy studies. Ann Intern Med.

[21] Renzulli M., Brocchi S., Cucchetti A., Mazzotti F., Mosconi C., Sportoletti C. (2016). Can current preoperative imaging Be used to detect microvascular invasion of hepatocellular carcinoma?. Radiology.

[22] Bi W.L., Hosny A., Schabath M.B., Giger M.L., Birkbak N.J., Mehrtash A. (2019). Artificial intelligence in cancer imaging: clinical challenges and applications. CA A. Cancer J Clin.

[23] Wang S., Liu Z., Rong Y., Zhou B., Bai Y., Wei W. (2019). Deep learning provides a new computed tomography-based prognostic biomarker for recurrence prediction in high-grade serous ovarian cancer. Radiother Oncol.

[24] Shin H-c, Roth H., Gao M., Lu L., Xu Z., Nogues I. (2016). Deep convolutional neural networks for computer-aided detection: CNN architectures, dataset characteristics and transfer learning. IEEE T Med Imag.

