# Supplementary material for: CT radiomics for prediction of microvascular invasion in hepatocellular carcinoma: A systematic review and meta-analysis
Source: Clinics (Sao Paulo). 2023 Aug 8;78:100264. doi: 10.1016/j.clinsp.2023.100264 (PMC10432601; doi:10.1016/j.clinsp.2023.100264)
Supplement: Supplementary file 1 [file mmc1.docx]

**CLINICS-D-23-00139_Supplementary Material**

**Table Supplementary Table 1** The radiomics quality score of each included study.

| **Study** | **Image protocol (2)** | **Multiple segmentations (1)** | **Phantom study (1)** | **Multiple time points (1)** | **Feature reduction (3)** | **Non radiomics (1)** | **Biological correlates (1)** | **Cut-off analyses (1)** | **Discrimination statistics (2)** | **Calibration statistics (1)** | **Prospective study (7)** | **Validation (5)** | **Gold standard (2)** | **Clinical utility (2)** | **Cost (1)** | **Open science (4)** | **Total (36)** |
| --- | --- | --- | --- | --- | --- | --- | --- | --- | --- | --- | --- | --- | --- | --- | --- | --- | --- |
| Yao W, et al.[7] | 1 | 1 | 0 | 0 | 3 | 1 | 0 | 1 | 1 | 0 | 0 | -5 | 2 | 2 | 0 | 0 | 7 |
| Yang Y, et al.[8] | 1 | 1 | 0 | 0 | 3 | 1 | 0 | 1 | 1 | 2 | 0 | 2 | 2 | 2 | 0 | 0 | 16 |
| Zhang W, et al.[9] | 1 | 1 | 0 | 0 | 3 | 0 | 0 | 1 | 1 | 1 | 0 | 2 | 2 | 2 | 0 | 0 | 14 |
| Liu SC, et al.[10] | 1 | 1 | 0 | 0 | 3 | 1 | 0 | 1 | 1 | 0 | 0 | 3 | 2 | 2 | 0 | 0 | 15 |
| Jiang YQ, et al.[11] | 1 | 1 | 0 | 0 | 3 | 1 | 0 | 1 | 1 | 0 | 0 | 2 | 2 | 2 | 0 | 0 | 14 |
| He M, et al. [12] | 1 | 1 | 0 | 0 | 3 | 1 | 0 | 1 | 1 | 2 | 0 | 2 | 2 | 2 | 0 | 0 | 16 |
| Zhang X, et al.[13] | 1 | 1 | 0 | 0 | 3 | 1 | 0 | 1 | 1 | 2 | 0 | 3 | 2 | 2 | 0 | 0 | 17 |
| Ni M, et al.[14] | 1 | 1 | 0 | 0 | 3 | 0 | 0 | 1 | 1 | 0 | 0 | -5 | 2 | 2 | 0 | 0 | 6 |
| Xu X, et al.[15] | 1 | 1 | 0 | 0 | 3 | 1 | 0 | 1 | 1 | 2 | 0 | 2 | 2 | 2 | 0 | 0 | 16 |
| Ma X, et al. [16] | 1 | 1 | 0 | 0 | 3 | 1 | 0 | 1 | 1 | 2 | 0 | 2 | 2 | 2 | 0 | 0 | 16 |
| Peng J, et al. [17] | 1 | 1 | 0 | 0 | 3 | 1 | 0 | 1 | 1 | 2 | 0 | 2 | 2 | 2 | 0 | 0 | 16 |

Value of parenthesis indicates the top score corresponding to each item.
